# The surgical treatment of upper cervical spine trauma in octogenerians

**DOI:** 10.1016/j.bas.2025.105863

**Published:** 2025-11-04

**Authors:** Mirza Pojskić, Miriam Bopp, Christopher Nimsky, Benjamin Saß

**Affiliations:** a Department of Neurosurgery, University of Marburg, Marburg, Germany; b Center for Mind, Brain and Behavior (CMBB) Marburg, Germany

**Keywords:** Upper cervical spine trauma, Dens fracture, Intraoperative computed tomography, Neuronavigation, Spine surgery in elderly, Octagenerians

## Abstract

**Introduction:**

Surgical treatment of upper cervical spine injuries in older patients is increasing in high-income countries and carries risks due to comorbidities, impaired general health, and osteoporosis.

**Research question:**

The aim of this study was to compare the surgical outcomes in patients aged 80–90 years who underwent surgical treatment for upper cervical spine fractures with those in a younger population.

**Materials and methods:**

All patients who underwent navigated dorsal stabilization of upper cervical spine trauma with automated intraoperative CT (iCT)-based registration at a single institution were retrospectively analyzed. Clinical and radiological outcomes were compared between patients over and under 80 years of age.

**Results:**

A total of 61 patients underwent dorsal stabilization after a C1/2 fracture. The mean age was 72.3 ± 16.4 years; 31 patients (50.8 %) were 80 years or older. Forty patients had an isolated C2 fracture, 18 patients had combined C1 and C2 fractures, and three patients had a simultaneous C2 and C3 fracture. All patients were registered intraoperatively and scanned intraoperatively to check the screw position. No screw misplacements occurred. The clinical and radiological results did not differ between the group of patients over 80 years of age and the group of patients under 80 years of age (p > 0.05).

**Discussion and conclusion:**

Surgical treatment of upper cervical spine injuries in elderly patients is as safe and effective as in younger patients. Despite higher mortality and longer hospital stays, surgical outcomes were comparable in terms of complications and fusion rates.

## Highlights

• Surgery for upper cervical trauma in octogenarians shows outcomes similar to younger patients despite comorbidities.
• Octogenarian and younger groups had comparable complication rates and similar clinical outcomes.
• Octogenarians had more comorbidities and higher ASA scores, yet maintained favorable surgical outcomes
• Thirty-day mortality was higher in octogenarians (6.4%) but due to pre-existing conditions, not surgery.

## 1 Introduction

Age itself is a recognized risk factor for complications in cervical spine surgery (Fehlings et al., 2013; Furlan et al., 2011; Patel et al., 2010). Older patients generally have more comorbidities, which, together with the duration of the operation, the type of procedure, and diabetes mellitus, are among the risk factors that influence the clinical outcome (Fehlings et al., 2012, 2013; Tamai et al., 2017).). Several studies define the geriatric population as older than 65 years and have shown that older patients did not have a significant risk of postoperative complications, even in the context of early and delayed surgery for cervical spine injuries (Shabo et al., 2022; Segi et al., 2025). Surgical treatment of upper cervical spine injuries in elderly patients over 80 years of age is increasing in high-income countries and carries risks due to comorbidities, poor general health, and osteoporosis. In patients over 80 years of age, a 2-year morbidity rate of up to 50 % has been reported, with complications occurring less frequently than 17 %, especially in patients with fractures. (Tan et al., 2019). Analysis of outcomes in 80-year-olds with subaxial cervical spine injuries showed that 30- and 90-day mortality rates were significantly higher in older patients due to multiple comorbidities, with an increased risk of medical complications but a comparable risk of surgical complications (El-Hajj et al., 2024).

**Table 1:** Summarizes characteristics of non-octagenerian and octagenerian group.

Characteristics	Non-octagenerian group (<80 years old)	Octagenerian group (80–90 years old)
Number of patients	30	31
Mean age	62.8 ± 12.1 years	84.3 ± 3 years
Gender	18 female	15 female
	12 male	16 male
Trauma etiology	Fall at home 12 patients Traffic accident 4 patients Fall outdoors 11 Fall from horse 1 Fall from stairs 2	Fall at home/in nursing home 20 patients Traffic accident 1 patient Fall outdoors 6 Fall from stairs 3
Fracture location	C2 fracture 19 patients	C2 fracture 21
	C1 and C2 fracture 10	C1 and C2 fracture 8
	C2 and C3 fracture 1	C2 and C3 fracture 2
Mean surgery time	185 ± 34 min	184.7 ± 56 min
Preoperative status	No preexisting neurological deficits	Eight patients required a wheelchair and/or walking aid due to their poor general condition or following a stroke.
ASA Score	12 patients ASA II	5 patients ASA III
	10 patients ASA III	26 patients ASA IV
	8 patients ASA IV	
Postoperative clinical outcome	29 patients could ambulate at pre-trauma level	28 patients could ambulate at pre-trauma level
	1 patient in locked-in syndrome due to traumatic brain injury	1 patient with severe traumatic brain injury was not mobile
		2 patients died within 30 days from surgery
Radiological outcome	Fusion in 29/30 patients	Fusion in 28/29 patients
Surgical complications	2 patients	2 patients
Medical complications	Pneumonia in 1 patient	Pneumonia in 5 patients Urinary sepsis in 1 patient
Postoperative treatment at ICU	4 patients	7 patients
Hospital stay	11.4 ± 8.2 days	15.4 ± 8.6 days
Comorbidities	Present in 22/30 patients, in 15 patients with multiple comorbidities: Obesity 4 Osteoporosis 2 Arterial hypertonus 7 Chronic obstructive pulmonary disease 2 Diabetes mellitus 4 Coronary heart disease 4 Atrial fibrillation under anticoagulation therapy 2 Malignancy 2 Infection disease (pneumonia, urinary infection, meningitis) 2	Present in 30/31 patients, all patients with multiple comorbiditiexs: Obesity 15 Osteoporosis 7 Arterial hypertonus 20 patients Chronic obstructive pulmonary disease 2 Diabetes mellitus 7 Atrial fibrillation under anticoagulant therapy 6 Coronary heart disease 4 AV block with pacemaker implantation 2 Malignancy 5 Infection disease (pneumonia, urinary infection, meningitis) 5 Renal insufficiency 2 Morbus Parkinson 2 Dementia 2
30 days mortality	None	2 patients
Discharge	7 patients neurological/orthopaedic rehabilitation 23 patients home discharge	18 patients geriatric rehabilitation 2 patients neurological//orthopaedic rehabilitation 5 patients nursing home 4 patients home discharge

Stabilization of the C1–C2 complex remains one of the most demanding procedures in spinal surgery, given the unique biomechanical environment and proximity to critical neurovascular structures. In elderly patients, odontoid and C1–C2 fractures are increasingly common due to rising life expectancy, osteoporosis, and low-energy falls, and are associated with considerable morbidity and mortality if left untreated. While non-operative management may be considered, recent cohort and registry studies have demonstrated that surgical stabilization offers superior union rates, improved pain relief, and better maintenance of functional independence in older patients, provided perioperative risks are appropriately managed (Fehlings et al., 2013; Schoenfeld et al., 2011; Robinson et al., 2018a, 2018b). Scheyerer et al. (2013) compared the three treatment modalities in elderly patients (anterior and posterior approach and conservative treatment) surgical treatment has shown higher survival rates compared to conservative and posterior approach has shown higher rate of fusion than anterior approach. Posterior C1–C2 fixation techniques, particularly screw-based constructs, have therefore gained traction even in octogenarians. Alongside these demographic shifts, technological advances have transformed intraoperative guidance. The integration of intraoperative CT imaging and neuronavigation allows three-dimensional trajectory planning and real-time verification of implant placement, thereby addressing the narrow safety margins of the upper cervical spine (Shin et al., 2012; Nottmeier and Foy, 2008). Contemporary series suggest that navigation-assisted C1–C2 fixation achieves higher screw placement accuracy and reduces the rate of neurovascular complications compared with conventional fluoroscopy, while minimizing the need for intraoperative revision (Costa et al., 2011). These developments highlight the importance of evaluating not only clinical outcomes but also the role of modern image guidance in improving the safety of C1–C2 stabilization in elderly populations. In 2019, a series comparing posterior stabilization in C2 fractures in patients undergoing navigated surgery with surface-matching based registration with patients undergoing automatic registration using intraoperative imaging was published (Carl et al., 2019). In the current study, we included all patients who underwent dorsal stabilization for upper cervical spine fractures using iCT-based automatic registration. The aim of this study was to compare the surgical outcomes in octogenarians (patients aged between 80 and 90 years) who underwent surgical treatment for C1/2 fractures with those in a younger population. Our hypothesis was that octogenarians would achieve comparable clinical and radiological outcomes to younger patients after dorsal stabilization surgery for upper cervical spine trauma.

## 2 Materials and methods

All patients who underwent navigated dorsal stabilization of upper cervical spine trauma (C1/2 fracture) with automatic intraoperative CT registration (iCT) at a single institution between 2016 and June 2025 were retrospectively analyzed. All patients signed informed consent for use of their data for research purposes. Due to the retrospective nature of the study and the fact that all data was collected as part of routine clinical practice, the ethics votum was considered unnecessary by the Ethics Committee our institution (RS 22/74, November 9, 2022). Clinical and radiological outcomes were compared between patients over and under 80 years of age. All patients with upper cervical fractures treated surgically by the neurosurgery department during the study period were eligible for inclusion. Patients managed conservatively within neurosurgery (n = 10) were excluded due to small numbers and heterogeneous follow-up. In our university hospital setting, case responsibility is shared between neurosurgery and orthopaedics/traumatology; thus, the majority of conservatively treated cases and a proportion of surgically managed cases are overseen by the orthopaedic/traumatology service. To ensure a homogeneous cohort and consistent follow-up, the present study was therefore limited to surgically managed patients treated under neurosurgery.

Age was dichotomized (<80 vs ≥ 80 years). Comorbidity burden was assessed with the Charlson Comorbidity Index (CCI) and ASA class. Blood loss (ml) and fracture type were recorded. Fractures were grouped as C2 type 2, C2 type 3, or combined C1/C2.

The treatment algorithm and indications for surgery at C1-C2 included the following.

a) The treatment algorithm for C1 fractures was performed according to the Gehweiler classification and the recommendations of the Spine Section of the German Society for Orthopaedics and Trauma Surgery (DGOU). Type I and type II injuries were treated conservatively. In type 3 injuries, the integrity of the transverse ligament of the atlas (LTA) was checked using magnetic resonance imaging. Unstable atlas fractures (type 3b and sagittal split fractures of type 4) were treated surgically (Ylönen et al., 2021; Mead et al., 2016; Kandziora et al., 2018; Laubach et al., 2022; Fiester et al., 2022).
b) Type I C2 fractures according to Anderson D'Alonzo (AA) were treated with a rigid cervical collar for six to twelve weeks, followed by X-ray and CT examination to check fusion. These fractures were not part of this study.
c) C2 fracture type II according to Anderson D'Alonzo: In young patients (<50 years) without risk factors for non-healing, a cervical collar was offered as a treatment option.; (Wallace et al., 2020), posterior C1-2 fusion according to Harms (Zhou et al., 2021; Carl et al., 2019; Ylönen et al., 2021) was recommended. In cases of minimal dislocation, stabilization with an anterior odontoid screw was offered as an alternative (Carvalho et al., 2019), however, in cases with risk factors for non-healing, such as age >50 years and neurological deficits or instability (Schroeder et al., 2015) posterior C1-2 fusion according to Harms technique (Harms and Melcher, 2001; Lvov et al., 2022; Schulz et al., 2011) was recommended. Older patients who were not ideal candidates for surgery due to their general condition and comorbidities were offered a hard neck brace for six to twelve weeks. However, due to the low probability of healing in advanced age, especially in patients who cannot tolerate immobilization, stabilization of C1-2 was preferred (Patel et al., 2010; Tashjian et al., 2006; Bransford et al., 2014; Dantas et al., 2025; Nouri et al., 2024). In type II fractures, in cases of high riding vertebral artery position and for anatomical reasons such as narrow C2 pars pedicles, where C1-2 stabilization could not be performed safely, occipito-cervical stabilization was performed (Arslan et al., 2024; Salunke and Karthigeyan, 2021; Klepinowski et al., 2025)
d) C2 type III Anderson-D'Alonzo fracture: unstable fracture, indication for dorsal C1-2 stabilization (Bourdillon et al., 2014)
e) The C2 hangman fracture was classified according to Levine and Edwards. Type I was treated conservatively, type II via an anterior approach, and type III either via C2 stabilization or posterior C1-3 stabilization if reduction was not possible after traction. (Scholz et al., 2018; Prost et al., 2019).
f) Atlantooccipital dislocation injuries and combined C1-2 injuries. Conservative treatment with halostabilization was offered in cases where there was no instability (atlanto-dental interval >5 mm and C2-3 angulation as a sign of instability). However, due to the increased morbidity and mortality associated with halostabilization in older patients, the increased rate of pseudarthrosis in type II odontoid fractures, and patient preference, surgical treatment was preferred. In cases where the fracture allowed C1-2 stabilization, posterior stabilization according to Harms was performed. In cases of significant displacement of the fracture fragments in C1 and C2, occipito-cervical stabilization was performed (Alves et al., 2020; Dagtekin et al., 2018; Hadley et al., 2002). In combined C1-2 fractures, an individual approach was chosen for each patient according to the anatomy of the pedicles and the displacement of the fracture fragments. Lateral mass screws were primarily used for occipito-cervical stabilization. If lateral mass screws could not be used for anatomical reasons or due to poor bone quality, pedicle screws were placed.

The surgical procedure to stabilize the posterior cervical spine involved placing the patient in the prone position with a positioning pillow and the head in a foam head restraint. A preoperative X-ray was taken with a C-arm to check the alignment. The skin incision was made in the midline with subperiosteal dissection of the cervical muscles. After exposing the posterior cranial fossa, the posterior arch of C1, C2, and the laminae of C3-7, a reference marker was usually placed on the spinous process of C7. An iCT scan was performed using a low-dose protocol with AIRO® CT for automatic registration, and accuracy was verified using four skin markers placed along the incision that were not part of the registration process. The screws were placed using a navigated technique. After screw placement, an iCT scan was performed to check the implant position.

All patients who underwent surgery between 2016 and 2022 were followed up for at least two years after surgery. Clinical outcome was considered favorable if patients had improved neurological status after surgery, or unchanged in case where there were no preoperative neurological deficits, as well as if the patients could ambulate following surgery and walk with or without walking aid. Radiological outcome was considered favorable if fusion, defined as radiographic evidence of successful bone bridging between adjacent vertebral segments in the follow up CT, indicating solid bony union following spinal fusion procedures.

Follow-up was updated through June 2025, extending the observation window to a median 4.5 years in 51/61 patients. Follow-up was ascertained via in-person clinic visits, structured telephone interviews (patient or caregiver), and review of electronic health records (operative notes, discharge summaries, radiology reports, and outpatient correspondence). Ten patients (6 octogenarians, 5 non-octogenarians) were lost to long-term follow-up and excluded from survival analyses but retained for perioperative outcomes when available.

Statistical analyses were performed using SPSS statistical software, version 20 (SPSS Inc., IBM, 1 Orchard Rd, Armonk, NY, USA), with a p-value of <0.05 considered statistically significant. Kolmogorov-Smirnov and Shapiro-Wilk tests were used to estimate normal distribution. Mean values and standard deviations (SD) were calculated, and a *t*-test was used to assess differences between mean values, while the Leven test for equality of variances was performed prior to the *t*-test to measure differences between standard deviations. In cases where a statistically significant difference between SDs was found, no *t*-test was performed. An independent *t*-test was performed to compare different means. Multivariable logistic regression was performed with favorable outcome as the dependent variable and age group as the main predictor, adjusting for CCI, ASA, and fracture type. Odds ratios (OR) with 95 % confidence intervals (CI) and p-values were reported. Sensitivity analyses including blood loss did not materially alter estimates. Analyses were performed using statsmodels (Python 3.11); original univariable tests were carried out in SPSS.

## 3 Results

### 3.1 General characteristics

A total of 61 patients underwent navigated dorsal stabilization following a C1/2 fracture. The average age was 72.3 ± 16.4 years; 31 patients (50.8 %) were 80 years of age or older (Table 1). There were 33 female (52.4 %) and 28 male patients.

Forty patients had an isolated C2 fracture, 18 patients had combined C1 and C2 fractures, and three patients had a simultaneous C2 and C3 fracture. Detailed characteristics of the cohort in chronological order from 2016 are added to Supplemental material.

Initially, conservative treatment with a rigid cervical collar was offered to patients with a type II C2 Anderson-D'Alonzo fracture, patients with old fractures with chronic subluxation (patient no. 18), and patients who refused surgical treatment (patient no. 25). Surgical treatment was primarily recommended for all unstable fractures, and surgery was also recommended for patients with risk factors for non-union (age >50 years, osteoporosis) even in cases of type II C2 Anderson-D'Alonzo fractures. Three of 61 patients in the current cohort initially received conservative therapy with a rigid cervical collar (patients 13, 18, and 25), but this did not result in fusion, so surgery was performed. In patient no. 19, a ventral discectomy, cage implantation, and plate osteosynthesis were performed due to a Hangman fracture LE II, but posterior stabilization C1-3 was performed due to lack of fusion. In patient no. 30, the screws were removed one year after surgery at patients request due to successful fusion. A total of three patients had metastatic spinal disease with metastases in C1 and C2, which were treated surgically due to instability after trauma.

### 3.2 Neurological deficits and concomitant injuries

No preoperative or postoperative neurological deficits due to fractures were observed in 60 patients. Eight patients required a wheelchair and/or walking aid due to their poor general condition or following a stroke in the over-80 age group. Four patients had severe concomitant traumatic brain injuries that required surgical treatment (patients 3, 7, 31, and 56). These patients were treated in the intensive care unit for an extended period after surgery and underwent surgery for a fracture of the upper cervical spine during their hospital stay. Patient No. 7 was in locked-in syndrome after a severe traumatic brain injury and a decompressive craniectomy; the other three patients with traumatic brain injury regained their ability to walk. Patient No. 12 had a chronic subdural hematoma and underwent elective surgery. Patients No. 25 and 47 had a small acute subdural hematoma after trauma, which was observed in the intensive care unit and monitored with CT without the need for surgical treatment.

Three patients had vertebral artery dissection (patients 40, 44, and 53), which was treated with aspirin and did not require endovascular intervention. There were no cerebral vascular ischemic events due to vertebral artery dissection.

Other concomitant injuries included lung contusion (patient no. 7), rib fracture and intestinal perforation (patient no. 13), metacarpal bone fracture with surgical treatment (patient no. 53) and radius fracture (patient no. 56).

### 3.3 Complications and mortality

All patients who underwent surgery between 2016 and 2022 were followed up for at least two years. Two patients died during hospitalization due to severe multiple organ failure (patients 10 and 60), one patient died six months after surgery from COVID-19 infection (patient 27), and one patient died two years after surgery for reasons unrelated to the surgical treatment (patient 8).

Patient number 10 was an 83-year-old female who underwent C1-2 stabilization for an unstable C2 fracture of type II according to Anderson D'Alonzo. The patient, who lived in a nursing home, suffered from dementia and New York Heart Association (NYHA) class III heart failure, atrial fibrillation, and chronic renal failure. The surgery was performed without intraoperative complications, and the patient was subsequently transferred to the general ward. The first five days after surgery were uneventful. On the sixth day after surgery, acute renal failure developed with rapid progression to heart failure and shock. The patient was transferred to intensive care unit and died on the seventh day after surgery due to decompensation of heart failure.

Patient number 60 is an 88-year-old male who fell at home. A CT scan revealed an unstable C2 fracture of type III according to Anderson D'Alonzo. The patient suffered from COPD, arterial hypertension, and diabetes mellitus, as well as chronic pulmonary insufficiency and recurrent pneumonia. After admission to the hospital, the patient was urgently intubated due to aspiration and the development of respiratory failure and pneumonia. CT scan following intubation has shown further dislocation of the C2 fracture. After stabilization of lung function and general condition, emergency occipito-cervical stabilization (C0-C3/4) with decompression was performed. After the operation, which was uneventful, the patient was transferred to the intensive care unit. His general condition was stable, allowing the patient to be extubated on the fourth day after surgery. On the sixth postoperative day, the patient developed progressive respiratory failure, requiring reintubation. However, the patient's pneumonia worsened and he succumbed to septic shock on the seventh day after the operation.

In non-octagenerian group, surgical complications occurred in two patients, and medical complications occurred in one patient who developed pneumonia which was successfully treated with antibiotics. Surgical complications occurred in two patients over the age of 80 (6.4 %), and medical complications such as pneumonia (one patient) and urinary tract infections (five patients) occurred in six patients (19.3 %). Two patients suffered a construct failure and had to undergo revision surgery (patient no. 20 with loosening of the occipital plate and patient no. 57 with failure of the C1-3 construct, which was subsequently revised and occipito-cervical stabilization performed). Two other patients underwent revision surgery due to wound healing deficits (patients 20 and 57).

### 3.4 Differences between octagenerian and non-octagenerian group

The group of patients over 80 years of age was significantly older than the group of patients under 80 years of age, had a higher incidence of patients with preoperative gait disorders and a significantly higher number of patients with one or more comorbidities, as well as a higher ASA score (p < 0.05). The 30-day mortality rate and length of hospital stay were higher in the group aged over 80 (p < 0.05). The rate of surgical complications and the clinical and radiological outcomes did not differ between the two groups (p > 0.05). Median follow-up was extended to 4.5 years (range 2.1–8.4) in 51 patients (83.6 %). Ten patients were lost to long-term follow-up (6 octogenarians, 5 non-octogenarians). At last follow-up, favorable outcomes were maintained in both groups concerning surgical clinical and radiological outcome.

Mean intraoperative blood loss was 406 ± 187 ml in patients <80 years and 514 ± 233 ml in octogenarians. While the difference was borderline with parametric testing (p = 0.053), non-parametric analysis confirmed significantly greater blood loss in the ≥80-year group (p = 0.042). Mean hospital stay was 11.6 ± 8.5 days in younger patients versus 15.2 ± 8.8 days in octogenarians. Similarly, although the *t*-test did not reach significance (p = 0.112), Mann–Whitney U testing indicated a significantly longer length of stay in octogenarians (p = 0.044). Seven patients required postoperative ICU treatment (3 octogenarians, 4 younger than 80 years). Mean intraoperative blood loss in this subgroup was 575 ml, which was higher than the cohort mean of 462 ml. The mean hospital stay was 14.9 days, likewise longer than the overall average of 13.5 days. Despite this increased perioperative burden, outcomes were largely comparable to the rest of the cohort: 5/6 ICU patients (83 %) had favorable outcomes, versus 43/54 (80 %) in the non-ICU group. Thus, ICU admission was associated with higher resource utilization but did not correspond to a substantially higher rate of unfavorable outcomes.

### 3.5 Multivariable analysis

In multivariable logistic regression adjusting for CCI, ASA, and fracture group, age ≥80 years was not associated with unfavorable outcome (OR 1.13; 95 % CI 0.22–5.73; p = 0.883). Neither CCI (OR 0.97; 95 % CI 0.78–1.21; p = 0.777) nor ASA (OR 0.44; 95 % CI 0.11–1.80; p = 0.251) independently predicted outcome. Compared with combined C1/C2 injuries, C2 type 2 (OR 0.69; p = 0.775) and C2 type 3 (OR 0.24; p = 0.227) were not significantly associated with outcome. Overall model fit was modest (Log-likelihood −26.9; Pseudo-R^2^ 0.111).

### 3.6 Illustrative cases

#### 3.6.1 Case 1

**Fig. 1:**
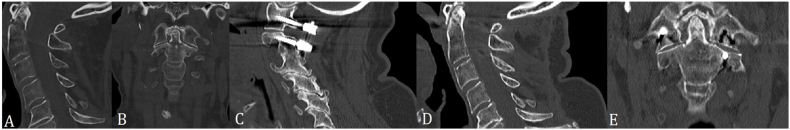
Preoperative CT scan of patient no. 28. The sagittal (A) and coronal (B) views show a type III AA fracture. The patient underwent navigated C1-2 stabilization using a navigated drill guide and implantation of navigated screws in C1 and C2 according to the Harms technique with iCT to check the implant position. One year after surgery, a CT scan was performed. C. The CT scan in the sagittal view shows screws with no signs of loosening, with signs of fusion in D. sagittal and E. coronal views.

**Fig. 2:**
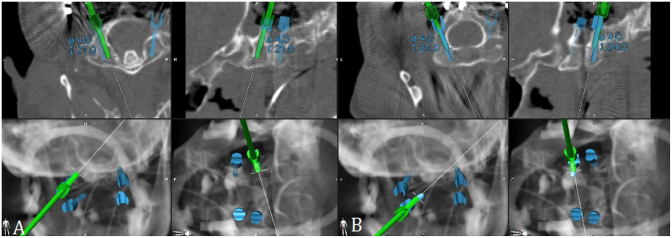
Intraoperative screenshots of patient no. 28 with navigated screw placement in A. lateral mass of C1 on the right side in axial and sagittal view with reconstruction and B. pars screw in C2 on the right side.

Patient No. 28 is a 74-year-old male who underwent C1-2 stabilization after a fall at home (Fig. 1, Fig. 2).

#### 3.6.2 Case 2

**Fig. 3:**
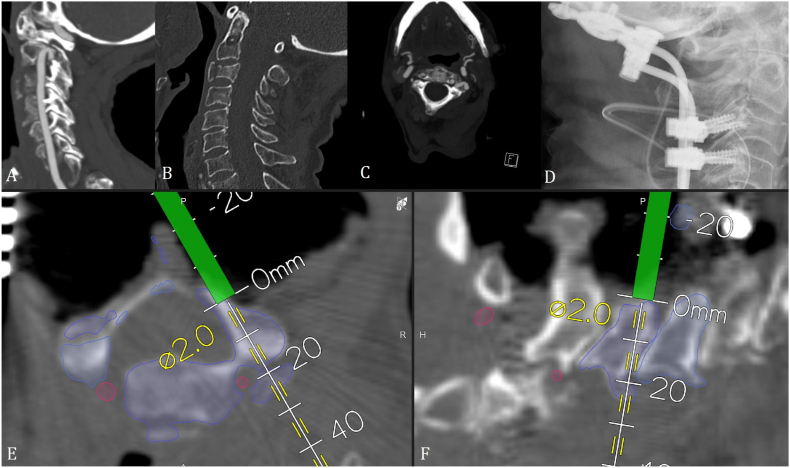
Patient No. 33. Preoperative sagittal A. CT angiography and B. CT of the cervical spine showing a Hangman's fracture with displacement and high-riding vertebral artery on the right side, as well as a C3 fracture. C. Axial CT angiography of the Hangman fracture. D. Postoperative X-ray after occipito-cervical stabilization. Intraoperative screenshots with drill guide for the implantation of a lateral mass screw in E. axial and F. sagittal view.

Patient No. 33, an 87-year-old female patient with a Hangman fracture LE III and C3 fracture, underwent occipito-cervical stabilization with lateral mass screws in C3 and C4 (Fig. 3).

#### 3.6.3 Case 3

**Fig. 4:**
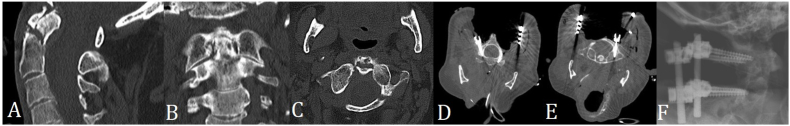
Patient No. 34. Preoperative CT in A. sagittal and B. coronal view shows a type II AA fracture, with C. axial view showing a type I Gehweiler fracture of the posterior arch on the left side. The patient underwent navigated C1-2 posterior stabilization. The iCT scan to check the implant position shows the correct placement of the D. C2 and E. C1 screws, which were inserted using the Harms technique. F. Postoperative lateral X-ray to check the alignment and screw position.

Patient No. 34, an 88-year-old female patient with AA 2 and Gehweiler I fracture who underwent C1-C2 stabilization. (Fig. 4).

## 4 Discussion

### 4.1 Novelty of the study

The present study extends the evidence on intraoperative CT–based navigation to one of the most challenging surgical populations: octogenarians with unstable upper cervical fractures. Intraoperative CT allows automatic 3D registration of the operative field, continuous tracking of instruments, and post-insertion verification of implants, overcoming the anatomical constraints and narrow safety margins at C1–C2. These features directly translate into improved implant accuracy and safety. Comparative series have consistently demonstrated superior accuracy of screw placement with iCT compared with conventional fluoroscopy (92–97 % vs ∼85–89 %) and lower rates of neurovascular breach and reoperation (Shin et al., 2012; Ito et al., 2008). Moreover, navigation reduces radiation exposure to the surgical team and, in many reports, does not prolong operative time (Härtl et al., 2013). In our cohort, the absence of screw misplacement and low reoperation rate likely reflect these advantages. Importantly, applying these benefits in octogenarians represents a novel contribution, as few prior studies have systematically examined advanced navigation in this age group. This underscores that the innovation of our work lies not only in confirming the technical superiority of iCT but also in demonstrating its safety and applicability in elderly patients, who are increasingly affected by upper cervical trauma.

### 4.2 Surgical vs. conservative treatment of upper cervical spine fracture treatment in elderly patients

The optimal treatment for patients with C2 fractures remains controversial. This is particularly true when deciding whether to perform surgery for complex injuries of the upper cervical spine, which occur occasionally, and in older patients with comorbidities and poor bone quality. However, there was broad consensus that surgical stabilization and fusion should be considered in cases of type 2 odontoid fractures with injury to the C1–2 segment and an ADI of more than 5 mm, a hangman's fracture with a C2–3 angulation of more than 11°, or failure of external immobilization. (Lee et al., 2024). In odontoid fractures, the most common fracture of the C2 vertebrae, anterior screw fixation is initially considered for type II fractures, and fusion of C1–2 is recommended if non-union is suspected or occurs. Hangman fractures are the second most common fractures. Many stable type I and II extension fractures can be treated with external immobilization, while the predominant type IIA and III flexion fractures require surgical stabilization. There are no results to prove that anterior or posterior surgery is superior, and the surgeon should decide on the surgical method after careful consideration according to the specific clinical situation**.** (Lee et al., 2024). In our cohort, posterior C1-2 stabilization according to Harms was preferred. In cases where C1-2 stabilization was not possible for anatomical reasons and due to fracture displacement, occipito-cervical stabilization was performed. In older patients in particular, posterior occipito-cervical fusion did not lead to an increase in the cervical disability index, although it reduced cervical rotation and range of motion. (Korovessis et al., 2020). In Anderson/D'Alonzo type 2 fractures, surgical treatment leads to solid fusion without an increased complication rate, even in older patients (Osterhoff et al., 2020, 2023). In osteoporosis, posterior techniques have a biomechanical advantage and can be considered standard (Osterhoff et al., 2020, 2023). The use of navigation to stabilize C2 fractures and fractures of the upper cervical spine facilitates the correct placement of implants and has been shown to reduce the complication rate, blood loss, and operating time (Hitti et al., 2017; Maes et al., 2020; Meyer et al., 2020; Singh et al., 2017). Compared to standard navigation, iCT-guided surgery enables greater navigation accuracy, shorter operating times, and a reduction in radiation exposure for patients and medical staff (Carl et al., 2019).

Epidemiological studies from the USA report that injuries to the upper cervical spine are on the rise and that these patients have an average age of 72.3 ± 19.6 years, are predominantly female and of Caucasian descent, and that these injuries are more frequently associated with falls and diabetes. (Futch et al., 2023, 2024). A recent national registry cohort analysis in Sweden reports that the incidence of C2 fractures increased from 3 to 6 per 100,000 between 1997 and 2014, mainly due to an increase in incidence in the geriatric subgroup (≥70 years) (Robinson et al., 2017). In older and very elderly patients with unstable type II C2 fracture, there is a tendency toward early surgical posterior stabilization, as the complication rate and mortality outweigh the benefits of early mobilization and lower morbidity (Issa et al., 2021). Shabo et al. (2022) reported in their evaluation of posterior stabilization in cervical spine trauma in a cohort of 153 patients that patients over 65 years of age had a complication rate of 14.5 % without an increased risk of complications or mortality compared to younger patients.

In a 2013 analysis of 322 patients by Chapman et al., a significant 30-day survival advantage and a trend toward improved long-term survival rates were demonstrated in surgically treated patients over the age of 65 with dens fractures compared to non-surgically treated patients. (Chapman et al., 2013). A systematic review of the treatment of geriatric odontoid found that short-term mortality was lower in patients who underwent surgical treatment than in patients who did not undergo surgery, and that there were no significant differences in complication rates. (Schroeder et al., 2015). In an analysis of the National Trauma Data Bank involving 6049 patients, of whom 2156 underwent surgery and 3893 were treated conservatively for a C2 fracture, the surgical group showed significantly lower mortality rates, longer hospital stays, and higher complication rates after propensity matching. This analysis included patients over the age of 65 as elderly individuals. (Jiang et al., 2024). This group had a higher discharge rate from rehabilitation centers, indicating a good prognosis. Studies by Fehlings et al. and Vaccaro et al. based on multicenter prospective cohort data have shown lower complication and mortality rates in the surgical cohort. (Fehlings et al., 2013; Vaccaro et al., 2013). Conservative strategies were associated with an increased likelihood of treatment failure, such as changes in the neck disability index, serious complications, and death, compared to patients who underwent surgery at an early stage. Advanced age, initial non-surgical treatment, and male gender were predictors of treatment failure regardless of whether conservative or surgical treatment was performed (Fehlings et al., 2013).

### 4.3 Complications and mortality

Surgical complications occurred in two patients over the age of 80 (6.4 %), and medical complications such as pneumonia and urinary tract infections occurred in six patients (19.3 %). Robinson et al., 2018a, 2018b. report that despite the frailty of older patients, morbidity with external immobilization of the cervical spine using a rigid neck collar is higher than surgical morbidity, even in patients over 80 years of age. In this study, non-surgical treatment was recommended only for patients over 88 years of age. A study by Issa et al. investigated the mortality and morbidity of patients over 90 years of age who had undergone surgery for an odontoid fracture. (Issa et al., 2021). There were no deaths in hospital, the average length of stay was 13.9 days, and the average intensive care treatment lasted 1.9 days. However, 33 % of patients died during the 26-month follow-up period. The complication rate was low at 7 %, which is comparable to our results. Mi Ryang et al. (2016) reported satisfactory results in high-risk patients with traumatic C2 fractures aged over 80 years. The results of posterior stabilization in a group of 50 patients with an average age of 87 years were evaluated, with 72 % experiencing preoperative, 14 % operative, and 50 % postoperative medical complications. One-third of patients in this cohort developed pneumonia after surgery. (Ryang et al., 2016).

Findings on longer mean hospital stay and greater blood loss in octagenerian group suggest an increased perioperative burden in the very elderly despite comparable surgical outcomes.In our cohort, two patients, i.e., 6.4 % of those over 80 years of age, died during hospitalization. Hospital mortality in a cohort of 891 patients with an average age of 78 years was 5.1 %, with no differences between treatment groups receiving Halovest, anterior or posterior stabilization for odontoid fractures. Male gender and a Charlson comorbidity index of ≥3 were the most important predictors of mortality. (Honda et al., 2023). Chan et al. (2019) reported on 1-year mortality in 70 patients over 65 years of age with odontoid fractures and identified the Charlson comorbidity index (CCI) and the modified frailty index (mFI) as predictors of 1-year mortality, which was 35 %. The 30-day mortality rate in older patients with odontoid fractures was reported to be 6 % (Ryang et al., 2016)to 14 % (Chan et al., 2019). A systematic review by Jubert et al. (2013) reported that in a group of 857 patients, 57 % of whom underwent surgery, the mortality rate was 9.2 % and the complication rate was 15.4 %.

### 4.4 Use on intraoperative imaging and neuronavigation for upper cervical spine trauma

Our findings build upon our previous single-center experience directly comparing standard navigation with intraoperative CT (iCT)–based navigation in upper cervical trauma (Carl et al., 2019). That study demonstrated significantly higher accuracy of screw placement and shorter operative times when iCT was used, with no screw misplacements in the iCT group compared to deviations observed under conventional navigation. Based on these results, iCT navigation was adopted as the standard of care in our department, and all subsequent cases—including those in the present series—were performed with CT-based navigation. Notably, the patients from the earlier publication who underwent iCT-assisted fixation are also included in the present cohort, thereby ensuring continuity of experience and demonstrating reproducibility of outcomes across an expanded patient population. The current work extends those findings to the particularly vulnerable octogenarian subgroup, confirming that the technical advantages of iCT navigation translate into safe and reliable outcomes even in very elderly patients.

Intraoperative CT (iCT)–based navigation provides automatic 3D registration and continuous instrument tracking at C1–C2, addressing the demanding anatomy and narrow safety margins of C2 pedicles/pars. Contemporary comparative data in traumatic atlantoaxial fixation show higher screw-placement accuracy with iCT versus fluoroscopic guidance. In a 78-patient comparison of atlantoaxial screw placement for traumatic instability, iCT achieved ∼97 % accuracy versus ∼89 % with fluoroscopy (p = 0.02), with fewer relevant breaches on post-implant 3D control scans (Gierse et al., 2024). These findings align with prior work in upper cervical fixation reporting >92–97 % accuracy using 3D navigation and iCT and reduced malposition-related revisions compared with freehand/fluoroscopy (Hagan et al., 2022). Beyond accuracy, iCT navigation reduces radiation exposure to the surgical team (at the trade-off of higher patient dose), and can streamline workflow by reducing reliance on repeated fluoroscopic shots; systematic and cohort studies demonstrate improved accuracy without longer operative time in many settings (Mendelsohn et al., 2016). Experimental and clinical reports comparing navigation platforms similarly indicate that modern CT systems with dose modulation can keep patient radiation within acceptable bounds while maintaining high precision (Baumgart et al., 2023). In our series, the absence of screw misplacement and low reoperation rate likely reflect these advantages of iCT-based automatic registration over conventional fluoroscopy.

### 4.5 Cost effectiveness

Economic considerations are increasingly relevant in the surgical management of elderly patients with upper cervical fractures. In our hospital system, intraoperative imaging and navigation are reimbursed within complex DRG structures, which offsets much of the additional cost associated with advanced intraoperative technology. Although octogenarians in our series had longer hospital stays and rehabilitation courses, this was not associated with increased readmissions, and perioperative complication rates were not higher than in younger patients. Blood loss was modestly greater in the elderly group. Importantly, cost-effectiveness analyses in this context are difficult to generalize, as they are strongly influenced by local healthcare systems, reimbursement models, and institutional resources. Previous studies suggest that navigation and intraoperative CT can be cost-effective when considering the potential reduction in reoperation rates and complications (Härtl et al., 2013; Dea et al., 2016). Cost savings are achieved if use of intraoperative CT prevents patients from requiring a return to the operating room (Hecht et al., 2011). Further prospective work is needed to validate these economic findings specifically in octogenarian populations.

### 4.6 Limitations

The limitations of this study include the small number of patients and its retrospective nature, as well as the lack of a propensity matching analysis and data on blood transfusions between the two groups. However, based on the experience of a single center and the unique treatment conditions, conclusions can be drawn about the differences between the two groups. We acknowledge the limited sample and low event rate; a post-hoc calculation indicates <80 % power to detect small between-group differences in complications and fusion. While our cohort is modest, it represents a homogeneous population treated with standardized techniques, reducing confounding variables. Small sample size, low event rate, retrospective design without a non-operative control, and inability to collect patient.reported outcomes limit inference; multivariable modeling was under-powered for small effect sizes Another limitation is that the conclusions of this study can only be applied to patients who did not have spinal cord compression or severe neurological impairment due to a fracture, although early surgery may have prevented the occurrence of these deficits, especially in the group of patients over 80 years of age and in a selected subgroup of patients with low compliance and dementia. Possible selection bias in interpretation of results is warranted in octagenerian group of patients lost to follow up, since these patients have higher mortality risk. higher mortality in octogenarians contradicts the conclusion of safety, however the two deaths in octogenarians were attributed to pre-existing conditions, not surgical complications, supporting the safety of the procedure itself.

## 5 Conclusion

Surgical treatment of upper cervical spine injuries in older patients is just as safe and effective as in younger people. Although longer hospital stays and higher mortality rates have been demonstrated in comparison to patients under 80 years of age, the clinical and radiological results, with a similar complication rate. After risk adjustment for comorbidity (CCI), ASA class, and fracture type, age ≥80 years did not independently predict outcome following navigated posterior fixation for upper cervical fractures. These findings support offering surgery to appropriately selected octogenarians.

## Ethics approval and consent to participate

This study was conducted in accordance with the principles of the 1964 Declaration of Helsinki. All patients gave their written consent for their data to be used for research and publication purposes. Ethical was waived by the local Ethics committee of University hospital Marburg, Philipps University, Az: RS 22/74, from November 9, 2022, in view of the retrospective nature of the study and all the procedures being performed were part of the routine care.

## Funding

No funding was received for conducting this study.

## Declaration of competing interest

All cases were operated using iCT-based navigation with BrainLab Munich Germany software. Christopher Nimsky and Miriam Bopp serve as scientific consultant for BrainLab. The other authors declare that they have no known competing financial interests or personal relationships that could have appeared to influence the work reported in this paper.
