# Supplementary material for: The surgical treatment of upper cervical spine trauma in octogenerians
Source: Brain Spine. 2025 Nov 4;5:105863. doi: 10.1016/j.bas.2025.105863 (PMC12657409; doi:10.1016/j.bas.2025.105863)
Supplement: Multimedia component 1 [file mmc1.docx]

Table 1. Characteristics of the cohort

| Patient Number | Age | Gender (F= female, M=male) | Fracture:  C1 Fracture Gehweiler I-IV  C2 Fracture: Anderson D´Alonzo AA 1-3  Hangman Levine/Edwards I-III LE 1-3 | Surgery  C1=Atlas, C2=Dens, PS=pedicle screws; otherwise lateral mass screws |
| --- | --- | --- | --- | --- |
| 1 | 70 | F | AA 3 | C1-2 |
| 2 | 67 | M | AA3, Gehweiler I | C1-2 |
| 3 | 83 | M | AA 3 | C1-2 |
| 4 | 77 | F | AA 3 | C1-3 |
| 5 | 78 | M | AA 3 | C1-2 |
| 6 | 80 | M | AA 2 | C1-2 |
| 7 | 24 | M | AA 2 | C1-2 |
| 8 | 68 | F | AA 3 | C1-3 |
| 9 | 89 | F | AA 2, Gehweiler 3a/Jefferson burst fracture | C1-2 |
| 10 | 83 | F | AA 2 | C1-2 |
| 11 | 87 | F | AA 3 | C1-2 |
| 12 | 80 | M | Hangman Fracture , LE III | C2 |
| 13 | 43 | F | AA 2 | C1-2 |
| 14 | 80 | M | AA 3, Gehweiler I | C1-3 |
| 15 | 70 | M | Metastasis in C2 with complex fracture following trauma | C1-2 |
| 16 | 85 | M | Metastasis in C2 with complex fracture following trauma | Occiput-HW3/4 |
| 17 | 89 | F | AA 3, Gehweiler 3a/Jefferson burst fracture | Occiput-C2-3 |
| 18 | 63 | F | Subluxation C1/2 due to C1 and C2 deformity fracture due to rheumatoid arthritis | C1-2 |
| 19 | 35 | F | Hangman Fracture, LE II | C1-3 following fusion failure after ventral discectomy, cage and plate C2-3 |
| 20 | 82 | M | AA 3 | Occiput-C3-4-5 |
| 21 | 44 | M | AA 2 | C1-2 |
| 22 | 67 | M | AA 3 | HW1-3 |
| 23 | 62 | M | AA 2, Gehweiler I | C1-2 |
| 24 | 81 | F | AA 3 | C1-2 |
| 25 | 82 | F | AA 3 | C1-2 |
| 26 | 89 | M | AA 2, Gehweiler I | Occiput-C2-3 |
| 27 | 81 | M | AA 3 | C1-3 |
| 28 | 74 | M | AA 2 | C1-2 |
| 29 | 85 | M | AA 3 | Occiput-C3-4 |
| 30 | 55 | M | AA 2 | C1-2 |
| 31 | 78 | M | AA 2 | C1-2 |
| 32 | 72 | F | AA 2 | C1-2 |
| 33 | 87 | F | Hangman Fracture LE III and C3 fracture | Occiput-C3-4 |
| 34 | 88 | F | AA 2, Gehweiler I | C1-2 |
| 35 | 79 | F | AA 2, Gehweiler I | C1-2 |
| 36 | 83 | F | AA 2 | Occiput – C3-4 |
| 37 | 40 | M | Hangman fracture LE II, Gehweiler 3a/Jefferson burst fracture | Occiput-C3-4-5 |
| 38 | 90 | F | AA 2 | C1-3 |
| 39 | 90 | F | AA 2 | C1-2 |
| 40 | 84 | F | LE III | Occiput-C3-4 |
| 41 | 83 | F | AA 2 | C1-2 |
| 42 | 81 | F | AA 3 | C1-3 |
| 43 | 88 | F | Hangman LE III and C3 Fracture | C1-4 PS |
| 44 | 54 | F | AA 3 | C1-2-3 |
| 45 | 80 | M | AA3, Gehweiler I | Occiput-C3-4 |
| 46 | 83 | M | AA 2, Gehweiler I | C1-2 |
| 47 | 87 | M | AA 3, Gehweiler I | Occiput-C3/4/5 |
| 48 | 57 | M | Hangman Fracture LE III | C1-3 |
| 49 | 61 | F | AA 3, Gehweiler 3a/Jefferson burst fracture | C1-3 |
| 50 | 82 | F | AA 3 | C1-3 |
| 51 | 71 | F | AA 3 | C1-3 |
| 52 | 82 | F | AA 2 | C1-2 |
| 53 | 79 | F | AA 3 | C1-2 |
| 54 | 66 | F | AA 2 | C1-2 |
| 55 | 83 | F | AA 2, Gehweiler I | Occiput-C3-4 |
| 56 | 64 | F | AA 3, Gehweiler IV | C1-2-4 |
| 57 | 68 | M | AA 3 | C1-3 |
| 58 | 79 | M | AA 3, Metastasis in C1 and C2 with fracture following trauma | C1-3 |
| 59 | 58 | F | LE II | C1-3 |
| 60 | 88 | M | AA 3 | Occiput-C3-4 |
| 61 | 66 | F | AA 3 | C1-2 |
